# Impact of abdominal drainage systems on postoperative complication rates following liver transplantation

**DOI:** 10.1186/s40001-015-0163-z

**Published:** 2015-08-21

**Authors:** Sascha Weiss, Franka Messner, Marcus Huth, Annemarie Weissenbacher, Christian Denecke, Felix Aigner, Andreas Brandl, Tomasz Dziodzio, Robert Sucher, Claudia Boesmueller, Robert Oellinger, Stefan Schneeberger, Dietmar Oefner, Johann Pratschke, Matthias Biebl

**Affiliations:** Department of Visceral, Transplant and Thoracic Surgery, Medical University Innsbruck, Anichstrasse 35, 6020 Innsbruck, Austria; Department of General, Visceral and Transplantation Surgery, Charité Universitätsmedizin Berlin, Augustenburger Platz 1, 13353 Berlin, Germany

**Keywords:** Liver transplantation, Drainage systems, Postoperative complications, Open-circuit drainage, Closed-circuit drainage

## Abstract

**Background:**

Depending on the extent of surgery, coagulation status and the number of anastomoses, drains are routinely used during liver transplantation. The aim of this study was to compare different drain types with regard to abdominal complication rates.

**Methods:**

All consecutive full-size orthotopic liver transplantations (LTX) performed over a 7-year period were included in this retrospective analysis. Abdominal drain groups were divided into open-circuit drains and closed-circuit drains. Data are reported as total number (%) or median (range); for all comparisons a *p* value <0.05 was considered statistically significant.

**Results:**

A total of 256 LTX [age 56.89 (0.30–75.21) years; MELD 14.5 (7–40)] was included; 56 (21.8 %) patients received an open-circuit Easy Flow Drain (Group 1) and 200 (78.2 %) a closed-circuit Robinson Drainage System (Group 2). For Groups 1 and 2, overall infection rates were 78.6 and 56 % (*p* = 0.001), abdominal infection rates 50.82 and 21.92 % (*p* = 0.001), yeast infection rates 37 and 23 % (*p* = 0.02), abdominal bleeding rates 26.78 and 17 % (*p* = 0.07), biliary complication rates 14.28 and 13.5 % (*p* = 0.51), respectively.

**Conclusions:**

In this retrospective series, open-circuit drains were associated with more abdominal complications, mainly due to intraabdominal infections, than were closed-circuit drains.

## Background

For centuries, body cavities have been routinely drained. Prophylactic drains remove postoperative fluid accumulations that have the potential to become infected and that function as an important factor in detecting early complications such as postoperative hemorrhage and anastomosis leakage. The benefits of drain insertion make this practice widely accepted and performed. Nevertheless, in general surgery, especially in gastrointestinal and liver surgery, we have observed increasing evidence to show that intraabdominal drainage following surgical procedures is unnecessary [1–3]. Several studies have failed to show advantages for drainage insertion after hepatic resection, especially in patients with normal livers [4–6]. Little has been published about the need for, usefulness, safety or complication rate of drain insertion following liver transplantation. To date, only less recent and very rare publications have dealt with this topic [7–11]. Despite the lack of information and evidence, most transplant centers use intraabdominal drains after this high-end surgical intervention and remove or close the drains within 5 days after surgery. Despite the possibility to totally eliminate intraabdominal drain insertion after liver transplantation, the question remains as to what kind of intraabdominal drain is associated with lower complication rates. While intraabdominal drains generally function without active suction, they differ mainly with regard to internal diameter, mechanism of fluid transportation (tube versus capillary system) and how the drains are connected to the collection system. The current literature makes no recommendation on whether to use open-circuit or closed-circuit drainage systems after liver transplantation. We here retrospectively analyze the use of a closed-circuit silicone drain and an open-circuit capillary drain with regard to appearance of postoperative abdominal complications after liver transplantation.

## Methods

We conducted a retrospective review of all consecutive full-size orthotopic liver transplantations (LTX) with primary closure of the abdomen performed over a 7-year period. Abdominal drain groups were divided into open-circuit silicone drains (Easy Flow Drain, Web-Sil Drain Easy Flow, 12 mm, Websinger, Wolkersdorf, Austria; Group 1) and closed-circuit silicone drains (Robinson Drainage System, 21 French, Dahlhausen, Cologne, Germany; Group 2).

Demographic and clinical data were recorded pre- and intraoperatively, as well as during the standardized postoperative follow-up at 3, 6, 12, 24 and 36 months. Follow-up was obtained in 100 % of patients.

Primary study endpoint was the infection rate at end of follow-up. Secondary study endpoints were surgery-related morbidity, bleeding, reoperation and biliary complication.

All patients were treated after granting written informed consent. Due to the retrospective nature of the study, the local institutional review board issued a waiver for IRB approval of this study protocol. Data are reported as totals (%) or median (range). Statistical analysis was performed using the SPSS 18.0 software (IBM, Michigan, USA), applying the Chi^2^ and the Mann–Whitney *U* test where appropriate. Differences of studied variables were identified by univariate analysis. Multivariate analyses were performed using stepwise Cox regression. *p* values <0.05 were deemed significant.

All consecutive patients receiving a full-size orthotopic liver transplantation from a deceased donor during the study period were included. The standard approach for liver transplantation was a bilateral subcostal incision with a midline extension. All incisions were closed 2-layered in a running fashion with looped 0 (peritoneum) and 1 (fascia) absorbable suture (PDS Covidien^®^). Orthotopic liver transplantation was performed by classical technique, as previously described [12]. No venovenous bypass was used in cases of retrohepatic caval resection. In only 4 % of the patients in Group 1 and 3 % of the patients in Group 2 was an orthotopic liver transplantation performed in piggyback technique. No patients with Roux-en-Y choledocho-jejunostomy were included in the study. The bile duct anastomosis was end-to-end or side-to-side with or without insertion of a T tube. In cases where a T tube was inserted, it was generally closed on postoperative day 5 after a contrast study demonstrated free run-off to the duodenum.

Postoperative surveillance included daily abdominal ultrasound exams in the first postoperative week and CT scans on clinical suspicion.

Immunosuppression consisted of induction therapy (Simulect^®^), followed by calcineurin inhibitors with or without mycophenolic acid maintenance. Steroids were tapered within 6 months post-transplant.

The drain was inserted before the abdominal wall was closed. The drains were routinely diverted from the right middle abdomen. In some cases where this was not possible the drains were placed in the left middle abdomen. In the open-circuit silicone drainage (Easy Flow) group, two drains were inserted through one incision. One intraabdominal end was placed in the retroperitoneal space, the second end near the hilum of the liver. Depending on coagulation state and bleeding tendency, one or two closed-circuit silicone drains (Robinson Drainage System) were inserted.

In the case of a biliary fistula that did not require surgical intervention, a lavage catheter was additionally inserted and continuous sterile lavage was initiated.

Our standard perioperative antibiotic regimen consisted of piperacillin/tazobactam. On transfer from the intensive care unit with running antibiotic and antifungal treatment, preoperative therapy was continued. Routine microbial analyses of the drainage fluid have been performed on postoperative day 3. The diagnosis of intraabdominal infections was made by standardized collection of drainage fluid with consecutive positive culture for pathogens (e.g. candida).

## Results

Two hundred and fifty-six consecutive patients were included in this retrospective study. Mean patient age in Group 1 (open-circuit drainage) and Group 2 (closed-circuit drainage) was 51.4 (12–68) and 52.6 (16–67) years, respectively. Group 1 comprised 12 female and 44 male patients (F:M ratio 1:3.67) and Group 2 comprised 47 female and 153 male (F:M ratio 1:3.26) patients. The main indications for liver transplantation were fatty liver cirrhosis due to alcohol, chronic viral hepatitis (HBV, HCV), PBC, sclerosing cholangitis, autoimmune hepatitis and acute liver failure, with malignant transformation of cirrhosis found in 24 (43 %) patients in Group 1 and 69 (35 %) in Group 2.

Relevant donor data and recipient comorbidities are listed in Table 1. Both groups were well matched with no significant differences found between the groups with regard to donor or recipient age (*p* = 0.7, *p* = 0.3), gender in donor and recipient groups (*p* = 0.4, *p* = 0.9), recipient BMI (*p* = 0.9) and MELD score (*p* = 0.3).

**Table 1:** Preoperative donor and recipient features

Parameters	Group 1 (open drainage)	Group 2 (closed drainage)	*p* value
Number of patients	56	200	
Donor age (years)	46.5 (24–74)	49.0 (15–76)	0.7 (ns)
Donor gender (m:f)	29:27	96:104	0.4 (ns)
Donor CMV-positive	34 (61 %)	110 (55 %)	0.2 (ns)
Donor ICU stay (days)	6 (1–16)	5 (1–14)	0.7 (ns)
Donor steatosis hepatis			
No	42 (75 %)	161 (80 %)	0.8 (ns)
Moderate	11 (20 %)	35 (17.5 %)	0.9 (ns)
Severe	3 (5 %)	4 (2 %)	0.8 (ns)
Recipient age (years)	51.4 (12–68)	52.6 (16–67)	0.3 (ns)
Recipient gender (m:f)	44:12	153:47	0.9 (ns)
Recipient CMV-positive	37 (66 %)	141 (70 %)	0.3 (ns)
CMV (donor+/recip.−)	12 (21 %)	38 (19 %)	0.8 (ns)
MELD score	17.0 (8–39)	16 (7–40)	0.3 (ns)
Body Mass Index	25.9 (17.4–35.4)	26.6 (18.5–36.2)	0.9 (ns)
Underlying disease			
Hepatitis B	5 (9 %)	13 (7 %)	0.4 (ns)
Hepatitis C	13 (23 %)	47 (24 %)	0.6 (ns)
Cryptogenic	2 (4 %)	12 (6 %)	0.4 (ns)
Primary biliary cirrhosis	1 (2 %)	9 (4.5 %)	0.3 (ns)
Biliary atresia	0	1 (0.5 %)	0.8 (ns)
Sclerosing cholangitis	1 (2 %)	5 (2.5 %)	0.6 (ns)
Autoimmune hepatitis	1 (2 %)	4 (2 %)	0.7 (ns)
Metabolic	0	11 (5.5 %)	0.1 (ns)
Acute liver failure	4 (7 %)	14 (7 %)	0.6 (ns)
Chronic alcoholic	29 (52 %)	84 (42 %)	0.5 (ns)
Presence of HCC^a^	24 (43 %)	69 (34.5 %)	0.1 (ns)
Child-Pugh score			
Child A	10 (18 %)	42 (21 %)	0.9 (ns)
Child B	32 (57 %)	110 (55 %)	0.9 (ns)
Child C	14 (25 %)	48 (24 %)	0.8 (ns)
Previous abdominal surgery	24 (43 %)	85 (42.5 %)	0.5 (ns)
Preoperative portal vein thrombosis	2 (4 %)	10 (5 %)	0.5 (ns)
Retransplantation	3 (5 %)	15 (7.5 %)	0.4 (ns)

Values are expressed as median (range)

*Group 1* open-circuit drainage (Easy Flow Drain), *Group 2* closed-circuit drainage (Robinson Drainage System)

^a^Hepatocellular carcinoma

Operative and perioperative data are shown in Table 2. Liver transplantation technique [retrohepatic caval resection (*p* = 0.5) vs. piggyback (*p* = 0.5)], type of choledocho-choledochostomy [end-to-end (*p* = 0.6) vs. side-to-side (*p* = 0.5)], use of T drains [with vs. no T tube (*p* = 0.1)], cold ischemia time (*p* = 0.7), need for blood transfusion (*p* = 0.3) and total operative times (*p* = 0.9) were comparable between the two groups.

**Table 2:** Recipient features peri- and intraoperatively and immunosuppression

Parameters	Group 1 (open drainage)	Group 2 (closed drainage)	*p* value
Number of patients	56	200	
Liver transplantation technique			
Piggyback technique	2 (4 %)	6 (3 %)	0.5 (ns)
Retrohepatic caval resection	54 (96 %)	194 (97 %)	0.5 (ns)
Bile duct anastomosis			
End-to-end	24 (43 %)	97 (49 %)	0.6 (ns)
Side-to-side	32 (57 %)	103 (51 %)	0.5 (ns)
T tube insertion (yes:no)	8:48	16:184	0.1 (ns)
Drain removal (postoperative day)	6 (4–13)	5 (5–11)	0.7 (ns)
Immunosuppression			
Tacrolimus and MMF	38 (68 %)	140 (70 %)	0.8 (ns)
Cyclosporin and MMF	11 (20 %)	52 (26 %)	0.8 (ns)
Simulect	53 (95 %)	185 (92 %)	0.9 (ns)
Cold ischemia time (min)	512 (212–954)	528 (229–1098)	0.7 (ns)
Operative time (min)	362 (238–712)	358 (360–643)	0.9 (ns)
Intraoperative blood transfusion (units)	4 (0–31)	3.5 (0–23)	0.3 (ns)
Intraoperative platelet transfusion (units)	2 (0–6)	2.5 (0–7)	0.4 (ns)
Intraoperative plasma transfusion (units)	6 (2–29)	7 (1–31)	0.6 (ns)

Values are expressed as median (range)

*Group 1* open-circuit drainage (Easy Flow Drain), *Group 2* closed-circuit drainage (Robinson Drainage System)

### Postoperative course and surgical morbidity after transplantation

Comparison of outcome and postoperative complication rates is shown in Table 3. Occurrence of primary non-function of the liver graft was similar in both groups (*p* = 0.6). Furthermore, no differences in postoperative ICU stay (*p* = 0.4) or length of hospital stay (*p* = 0.3) were detected. Vascular complications such as hepatic artery dissection (*p* = 0.4) or hepatic artery and portal vein thrombosis (*p* = 0.5, *p* = 0.6) were comparable in both groups. Bile duct complications such as bile duct leakage (*p* = 0.4) and stenosis (*p* = 0.1) were also comparable. The number of reoperations was significantly increased in the open-circuit drainage group (*p* = 0.04). In addition, we encountered significantly more intra-abdominal hematomas in Group 1 (*p* = 0.005). The occurrence of bleeding (defined as need for packed red blood cells after POD 1) showed a tendency to be higher in Group 1, but without statistical significance (*p* = 0.07).

**Table 3:** Outcome and surgical morbidity following liver transplantation

Parameters	Group 1, *n* = 56 (open drain)	Group 2, *n* = 200 (closed drain)	*p* value (univariate)	*p* value (multivariate)
Primary non-function	1 (2 %)	3 (1.5 %)	0.6 (ns)	–
Postoperative ICU stay (days)	5 (2–57)	4 (1–59)	0.4 (ns)	–
Postoperative hospital stay (days)	23 (11–108)	21 (13–98)	0.3 (ns)	–
Postoperative severe acute renal failure with dialysis	13 (23 %)	39 (19.5 %)	0.7 (ns)	–
Acute rejection	12 (21 %)	21 (10.5 %)	*0.03 (s)*	0.06 (NS)
Reoperation	31 (55 %)	82 (41 %)	*0.04 (s)*	0.07 (NS)
Bleeding	15 (27 %)	34 (17 %)	0.07 (ns)	–
Hematoma	21 (38 %)	39 (20 %)	*0.005 (s)*	*0.042 (S)*
Ascites	0	2 (1 %)	0.6 (ns)	–
Wound healing complication	8 (14 %)	18 (9 %)	0.2 (ns)	–
Neurological complication	4 (7 %)	17 (9 %)	0.5 (ns)	–
Hepatic artery dissection	1 (2 %)	1 (0.5 %)	0.4 (ns)	–
Hepatic artery thrombosis	1 (2 %)	2 (1 %)	0.5 (ns)	–
Portal vein thrombosis	0	2 (1 %)	0.6 (ns)	–
Bile duct leak	2 (3.6 %)	12 (6 %)	0.4 (ns)	–
Bile duct ischemia	0	1 (0.5 %)	0.6 (ns)	–
Bile duct stenosis	3 (5.4 %)	6 (3 %)	0.4 (ns)	–
Cholestasis	5 (9 %)	8 (4 %)	0.1 (ns)	–

Values are expressed as median (range)

Italic values indicate statistical significance of *p* value (*p* > 0.05)

*Group 1* open-circuit drainage (Easy Flow Drain), *Group 2* closed-circuit drainage (Robinson Drainage System)

### Infectious complications after liver transplantation

Postoperative infection rates are shown in Table 4. The overall infection rate was significantly higher in Group 1 (*p* = 0.001) and was related to an increased abdominal infection rate in this group (*p* = 0.001). While bacterial infections did not significantly differ between the two groups, the rate of candida infections was increased in Group 1 (*p* = 0.02). Other fungal infections (aspergillus *p* = 0.1) showed no differences between the two patient groups.

**Table 4:** Multivariate regression analysis of infection following liver transplantation after exclusion of non-significant parameters in univariate analysis

Parameter	Group 1, *n* = 56 (open drain)	Group 2, *n* = 200 (closed drain)	*p* value (univariate)	*p* value (multivariate)
Overall infection rate	44 (79 %)	98 (49 %)	*0.001 (s)*	*0.032 (S)*
Wound infection rate	9 (16 %)	21 (11 %)	0.2 (ns)	–
Cholangitis	2 (4 %)	3 (1.5 %)	0.3 (ns)	–
Gastrointestinal infection	1 (2 %)	6 (3 %)	0.5 (ns)	–
MRSA	1 (2 %)	0	0.2 (ns)	–
Clostridium	1 (2 %)	7 (3.5 %)	0.5 (ns)	–
Sepsis	1 (2 %)	10 (5 %)	0.3 (ns)	–
Oral candidiasis	1 (2 %)	2 (1 %)	0.5 (ns)	–
Central venous catheter infection	8 (14 %)	32 (16 %)	0.5 (ns)	–
Herpes simplex infection	14 (25 %)	47 (24 %)	0.5 (ns)	–
CMV infection	10 (18 %)	36 (18 %)	0.6 (ns)	–
Epstein-Barr virus	0	1 (0.5 %)	0.8 (ns)	–
CoNS	29 (52 %)	90 (45 %)	0.2 (ns)	–
ESBL-producing Enterobacteriacae	11 (20 %)	22 (11 %)	0.7 (ns)	–
Overall abdominal infection rate	28 (50 %)	45 (22.5 %)	*0.001 (s)*	*0.021 (S)*
*Candida albicans* infection rate (abdomen)	23 (41 %)	39 (19.5 %)	*0.02 (s)*	*0.036 (S)*
*Aspergillus* spp.	2 (4 %)	1 (0.5 %)	0.1 (ns)	–
Other	3 (5 %)	5 (3 %)	0.2 (ns)	–

Italic values indicate statistical significance of *p* value (*p* > 0.05)

*Group 1* open-circuit drainage (Easy Flow Drain), *Group 2* closed-circuit drainage (Robinson Drainage System)

No differences between the two groups were found with regard to positive cultures for MRSA (*p* = 0.2), clostridium infections (*p* = 0.5), oral candidiasis (*p* = 0.5), central venous catheter infections (*p* = 0.5), CoNS (*p* = 0.2), ESBL-producing Enterobacteriacae (*p* = 0.7), or viral infections such as herpes simplex virus infection (*p* = 0.5), CMV infection (*p* = 0.6) or Epstein-Barr virus infections (*p* = 0.8). Statistical analysis of all patients undergoing reoperation showed no significant difference in occurrence of abdominal fungal infections (Table 5).

**Table 5:** Abdominal fungal infections following liver transplantation in subgroups with reoperation

Parameter	Group 1, *n* = 31 (open drain)	Group 2, *n* = 82 (closed drain)	*p* value
Overall abdominal infection rate	6 (19.5 %)	19 (23 %)	0.64 (ns)
*Candida albicans* infection rate (abdomen)	4 (13 %)	15 (18 %)	0.71 (ns)

*Group 1* open-circuit drainage (Easy Flow Drain), *Group 2* closed-circuit drainage (Robinson Drainage System)

## Discussion

The type of abdominal drainage system to be used as standard procedure following orthotopic liver transplantation is rarely discussed today. Intuitively, the insertion of drainages seems critical, considering the often impaired coagulation status of the patients and the potential for leakage of the biliary anastomosis. Interestingly however, no comparative studies have evaluated the value of insertion of drains after liver transplantation. Sporadically, the use of no drains at all after liver transplantation has been reported [7–11, 13]. Nevertheless, this seems only anecdotal in highly selected patients, as most transplant centers use intra-abdominal drains after such an operation. Regarding liver resection surgery, there is however increasing evidence that sheer prophylactic insertion of drainage systems might be unnecessary or even associated with a higher complication rates, such as infection or bleeding [4–6]. Similar results have been published for other long-believed areas of certainty for intra-abdominal drainage such as colorectal and gastrointestinal surgery [2, 14–21].

With drain insertion being common surgical practice after liver transplantation, we aimed to investigate which drainage systems are associated with the lowest postoperative complication rates. While in the US, closed-circuit suction drainages such as a Jackson-Pratt drain are most widely used in liver transplant patients, in Europe, either open-circuit passive capillary drainages, or closed-circuit passive silicone drainages (typically 21 or 24 Charriere diameter) are widely used. To prevent erosion of intraabdominal structures both drainage systems are used without active suction. An advantage of the open circuit capillary drainage is the lower rate of obstruction compared to the closed circuit silicone drainage. This is one of the reasons for the broad application in conventional abdominal surgery. However, especially in the early postoperative period, these open-circuit drainages might have disadvantages in the transplant patient population.

Evaluation of vascular complications and biliary leakages showed comparable results for both groups in our study. We noticed no significant differences in occurrence of hepatic artery thrombosis, portal vein thrombosis or hepatic artery dissection between the study groups. Overall, in this series we observed very low vascular complication rates as compared with the current literature. Hepatic artery thrombosis, as most frequent arterial complication, occurs in 3–9 % of adult liver transplant recipients [32–34].

However, the results for reoperation after liver transplantation are different. In Group 1, 55 % of patients were reoperated, mostly due to hematoma; in Group 2 we reoperated 41 % of patients (*p* = 0.04). Reoperation was performed before removal of drains in 35 % of the patients in the group with open-circuit drains and in 41 % of the patients in the group with closed-circuit drains. The question remains whether open-circuit drainage systems are causally responsible for this. In case of reoperation due to hematoma, we rarely were able to identify an active source of bleeding. Nevertheless, the placement of open-circuit drainage systems would seem to be associated with much greater tissue injury. The amount of tissue damage to the abdominal wall observed for Easy Flow Drain insertion was approximately twice as much as for the Robinson Drainage System, and indeed was the suspected source of hematoma the abdominal wall at the drain entry site in several patients in the open-circuit drainage group. In addition, in this retrospective study, patients with open-circuit drains were more often reoperated overall than were patients with closed-circuit drains.

### Infectious complications after liver transplantation

Infection post LTX occurs in up to 80 % of patients and includes bacterial (70 %), viral (20 %) and fungal infections [35–38]. Risk factors for infection following liver transplantation may be divided into transplant-related factors, donor- and recipient-related factors. Risk factors for infection after liver transplantation include ischemia reperfusion injury, intraoperative blood/platelet and plasma transfusion, type of immunosuppression, rejection rate, prolonged duration of intensive care with or without mechanical ventilation of donor and recipient, postoperative acute renal failure with dialysis requirement, type of biliary reconstruction and retransplantation. Associated risk factors are quality of the graft, MELD score >30, malnutrition and immune status of the recipient [39–42]. Except for the occurrence of acute rejection, we observed no statistical difference with respect to the risk factors for infection.

Intriguingly, the overall rate of abdominal infection was significantly increased in the group with open-circuit drainage systems (*p* = 0.001). In particular, intraabdominal yeast infections were significantly more prevalent (*p* = 0.02). Infections at sites other than the abdomen were not significantly different between both groups.

The incidence of candida peritoneal involvement during postoperative intensive care ranged from 4 to 32 % [43, 44]. In liver transplant recipients, the incidence of fungal infection ranged from 7 to 42 % with *Candida* spp. and *Aspergillus* spp. as the pathogens most responsible for infection [50]. Pungpapong et al. reported a series of 950 liver transplantations with 108 (11 %) episodes of peritonitis [45], approximately 30 % caused by fungal infection. This gives a total of 3 % of invasive fungal infections. In contrast, the incidence of fungal infection in other studies appears higher. The colleagues obtained the ascitic fluid by diagnostic paracentesis. In the present and other published studies, ascites was obtained directly from the drainage or bag system under aseptic conditions. Even if we can assume iatrogenic contamination as compared to paracentesis, we observed a significant tendency for fungal colonization in the group of open-circuit drainage systems. *Candida* spp. are common colonizers of the human gastrointestinal and genitourinary tract and only a subgroup of the commensal flora [53]. While the infection rate observed when using the closed-circuit drainage systems is comparable to that of other publications, we report a significantly increased incidence of fungal infection with open-circuit drainage systems.

Summarizing, we can state, that liver transplant patient are more susceptible to develop bacterial, viral and, in comparison to other transplant recipients, in particular fungal infections. This susceptibility to infection is due to the complexity of the surgical procedure with involvement of the hepatobiliary system [40, 41, 54]. Our findings, which show an increased incidence of fungal infections with open-circuit drainage systems, seems plausible as this represents an artificial connection to the environment, which can more frequently facilitate retrograde contamination of the abdominal cavity. Still only few clinical data are published on this topic, so many questions remain unanswered. Randomized clinical trials are needed to further evaluate the safety and benefits of closed-circuit drainage systems.

## Conclusion

Based on the strong influence of fungal infections on mortality and morbidity after liver transplantation, we postulate a significant advantage for closed-circuit drainage systems to exclude a further risk factor.

## Abbreviations

LTX: liver transplantation
BMI: Body Mass Index
MELD: model for end-stage liver disease
ATG: anti-thymocyte globulin
HBV: hepatitis B virus
HCV: hepatitis C virus
PBC: primary biliary cirrhosis
ICU: intensive care unit
MRSA: methicillin-resistant staphylococcus aureus
CMV: cytomegalovirus
CoNS: coagulase-negative staphylococcus
ESBL: extended spectrum beta lactamase
HAT: hepatic artery thrombosis
PVT: portal vein thrombosis
